# Mechanism of Resistance in Mungbean [*Vigna radiata* (L.) R. Wilczek var. *radiata*] to bruchids, *Callosobruchus* spp. (Coleoptera: Bruchidae)

**DOI:** 10.3389/fpls.2017.01031

**Published:** 2017-06-20

**Authors:** Abdul R. War, Surya Murugesan, Venkata N. Boddepalli, Ramasamy Srinivasan, Ramakrishnan M. Nair

**Affiliations:** ^1^World Vegetable Center, South Asia, HyderabadIndia; ^2^World Vegetable Center, ShanhuaTaiwan

**Keywords:** mungbean, bruchids, biotic stress, breeding constraints, resistance

## Abstract

Mungbean [*Vigna radiata* (L.) R. Wilczek var. *radiata*] is an important pulse crop in Asia, and is consumed as dry seeds and as bean sprouts. It is an excellent source of digestible protein. Bruchids [*Callosobruchus chinensis* (L.) and *Callosobruchus maculatus* (F.)] are the important pests of mungbean and cause damage in the field and in storage. Bruchid infestation reduces the nutritional and market value of the grain and renders seeds unfit for human consumption, agricultural and commercial uses. These pests are controlled mainly by fumigation with highly toxic chemicals such as carbon disulfide, phosphene, and methyl bromide, or by dusting with several other insecticides, which leave residues on the grain, thus, threatening food safety. Some plant-based extracts have been found useful in controlling bruchids, but are not fully successful due to their short-term activity, rapid degradability, and potentially negative effect on seed germination. Although some wild sources of bruchid resistance in mungbean have been reported, which have been used to develop bruchid- resistant lines, undesirable genetic linkages threaten the proper exploitation of genetic diversity from wild germplasm into commercial cultivars. Further, biotype variation in bruchids has rendered some mungbean lines susceptible that otherwise would have been resistant to the pest. Host plant resistance is a cost-effective and a safe alternative to control bruchids in mungbean and is associated with morphological, biochemical, and molecular traits. These traits affect insect growth and development, thereby, reduce the yield losses by the pests. Understanding the defense mechanisms against insect pests could be utilized in exploiting these traits in crop breeding. This review discusses different traits in mungbean involved in defense against bruchids and their utility in pest management. We also highlight the breeding constraints for developing bruchid-resistant mungbean and how can these constraints be minimized. We further highlight the importance of supporting conventional breeding techniques by molecular techniques such as molecular markers linked to bruchid resistance.

## Introduction

Mungbean or green gram [*Vigna radiata* (L.) R. Wilczek var. *radiata*] is an important legume crop. It serves as a cash crop for farmers and is an excellent source of digestible protein of low flatulence. It is consumed as dry seeds and as bean sprouts. It is grown in tropical and sub-tropical regions mainly as part of cereal-based cropping systems. Mungbean is popular among farmers for its short life cycle and drought tolerance; nitrogen fixation in its root nodules in association with soil rhizobia allows it to thrive in N-deficient soils (Yaqub et al., 2010). The global annual production of mungbean is 3 million tons from more than 6 million hectares (Nair et al., 2013). India is the biggest producer of mungbean, with 3.5 million ha under cultivation and the production of 1.2 million tons (IIPR, 2011). Mungbean production is constrained by an array of destructive pests, a notable group of which are the storage pests. Among them, bruchids belonging to the genus *Callosobruchus* (Coleoptera: Bruchidae) are the most critical. These include *Callosobruchus chinensis* (L.) and *Callosobruchus maculatus* (F.) (Southgate, 1979; Talekar, 1988).

Although some reviews discuss bruchid resistance in legumes and other crops (Tripathy, 2016), none target the specific crop systems. In this review, we provide insight on different physical, biochemical and molecular mechanisms involved in the mungbean-bruchid interaction. These morphological and biochemical traits could form important markers for breeding bruchid-resistant mungbean and developing insect pest management programs for bruchids. This will help in reducing reliance on indiscriminate use of pesticides in controlling bruchids in storage. We further focus on the constraints faced by the breeders seeking to develop bruchid-resistant mungbean and ways to counter these challenges.

## Bruchids Infesting Mungbean and their Control

Out of about 1300 species of seed beetle in the family Bruchidae, 20 are recognized as pests, usually in stored legume seeds (Talekar, 1988). Four species, *C. maculatus* and *C. chinensis*, *Acanthoscelides obtectus* (Say) and *Zabrotes subfasciatus* (Boleman) are the major ones (Southgate, 1979). The *C. maculatus* and *C. chinensis* are the most destructive and attack almost all edible legumes, including mungbean, pigeon pea, black gram, cowpea, chickpea, and lentil, and are cosmopolitan in distribution, encompassing Australia and Oceania, Europe, Asia, Africa, and America (Rees, 2004). Bruchids are the most destructive pests of mungbean during storage and take a heavy toll on yield (Talekar, 1988). In mungbean, bruchid infestation occurs both in the field and in storage. However, storage losses are heavy and sometimes total losses occur within 3–6 months (Somta et al., 2007, 2008; Duan et al., 2014; Tripathy, 2016). Bruchid infestation in mungbean results in weight loss, low germination, and nutritional changes in seeds, thereby reducing the nutritional and market value, rendering it unfit for human consumption, agricultural and commercial uses (Talekar, 1988; Rees, 2004; Oke and Akintunde, 2013; Duan et al., 2014). Infestation by bruchids leads to an increase in trypsin inhibitor activity by 25%, saponin by 16%, and phytic acids by 46%, thus, making the seeds unfit for consumption (Modgil and Mehta, 1997). Bruchids are controlled by treating stored seeds with carbon disulfide, phosphine, or methyl bromide, or by dusting with several other insecticides. These chemicals are highly toxic and environmentally undesirable, and pose a threat to food safety. Although some plant-based extracts such as soy oil, maize oil, neem oil, hot pepper powder, custard apple extracts, and banana plant juice have been found useful in controlling bruchids (Koona and Dom, 2005; Swella and Mushobozy, 2007), they are slow in action, are easily degradable, and can affect seed germination (Yusuf et al., 2011). Botanical extracts also affect non-target organisms to some extent (Sharma et al., 2012). The use of dust and wood ashes in spaces between seeds provides some control of bruchids. However, these methods are not highly effective and are too expensive and laborious for resource-poor farmers (Tripathy, 2016). Breeding programs to incorporate host plant resistance against bruchids combined with good agronomic practices can address storage problems in mungbean and ensure that more of this nutritious legume will be available to enrich diets of the malnourished.

## Life Cycle and Ecology of Bruchids

The life cycle and ecology of both *C. maculatus* and *C. chinensis* are similar. The initial infestation originates in the field. The eggs are firmly glued to pods and seeds. In the field, eggs are laid on pods and in storage directly on the seeds. Bruchids lay 1–3 eggs/seed, and greater seed size accommodates a larger number of eggs. Yellow colored seeds are preferred to green or black seeds for oviposition and bruchid development. Seed coat or testa plays an important role in oviposition stimulation (Asian Vegetable Research and Development Center [AVRDC], 1988). Hatching of the eggs takes place after 6 days of oviposition (Devi and Devi, 2014). The larvae penetrate into the seed and develop inside it. Larvae excavate an emergence tunnel to the seed surface, forming a translucent window in the seed coat for the adult emergence. Although adults do not feed on seeds, they may feed on pollen and flower nectar in the field (Brier, 2007). The adult *C. maculatus* males and females both have an average life span of 7 days under laboratory conditions and only a few can survive more than 2 weeks (Fatima et al., 2016). The oviposition by *C. maculatus* reaches a peak within 2 days after the commencement of oviposition and declines over time (Barde et al., 2014). Each female lays about 100 eggs. The life cycle of both species is about 28–30 days at 30°C and 70% relative humidity (Raina, 1970).

The bruchids use vibrations from the egg laying substrates for oviposition (Messina and Renwick, 1985; Messina, 2004; Guedes and Yack, 2016). Bruchids do not feed during the brief adult stage and reproduction depends mainly on the resources the insect accesses during larval stages from a single seed, with severe competition from other larvae (Messina, 2004; Guedes et al., 2007). To avoid fierce larval competition in future—which may occur even inside the seeds, allowing only the emergence of larvae with good fitness—females lay a large number of eggs, depending on the substrate quality that the female perceives (Messina, 2004; Guedes et al., 2007; Guedes and Yack, 2016). A good quality substrate will lead to good larval fitness and a successful population build-up. Female bruchids have the ability to check the host suitability, deposit proper egg load, and discriminate the eggs laid by other females on a seed (Oshima et al., 1973; Messina, 2004; Guedes and Yack, 2016). The females use various tactile, chemical, and physical cues to choose suitable egg-laying substrate. These include multiple sensory modalities, egg-marking pheromone, and larval feeding vibrations from the seed (Oshima et al., 1973; Ignacimuthu et al., 2000; Tanaka, 2000; Guedes and Yack, 2016). However, the chemical cues last only for a few weeks and are influenced by environmental factors (Tanaka, 2000). The vibration cues and egg-marking pheromone play an important role in reducing egg density on a seed and in turn, minimizing larval competition.

## Sources of Bruchid Resistance in Mungbean

Only a few sources of mungbean resistant to bruchids have been found. Initially, TC1966, a wild mungbean [*V. radiata* var. *sublobata* (Roxb.) Verdc.], collected in Madagascar, showed complete resistance to *C. maculatus* and *C. chinensis* (Fujii and Miyazaki, 1987; Fujii et al., 1989). The World Vegetable Center screened about 100 *V. radiata* var. *sublobata* accessions against *C. chinensis*, and all possessed resistance against the pest. In these accessions, only 10–20% seeds had bruchid eggs and the seeds are rough textured with hard testa (Asian Vegetable Research and Development Center [AVRDC], 1990a). F_2_ derived from crosses between bruchid-resistant mungbean and high yielding mungbean breeding lines, bruchid-resistant TC1966 and high-yielding but susceptible mungbean lines showed that the resistance in F_2_ population was moderate and genetically controlled (Asian Vegetable Research and Development Center [AVRDC], 1990b). The resistance in TC1966 is controlled by a single dominant gene, *Br* (Kitamura et al., 1988; Fujii et al., 1989). Later, several resistant lines were developed successfully using TC1966 as the source (Tomooka et al., 1992; Watanasit and Pichitporn, 1996). However, wild species have been reported to have harmful anti-nutrients for humans and could possess unwanted wild characters due to linkage drag. For example, mice fed with artificial diets containing bruchid-resistant lines having wild mungbean TC1966 as the resistance source showed unwanted changes in blood biochemical values (Miura et al., 1996). Pod shattering has also been reported in a commercial variety developed from TC1966 (Watanasit and Pichitporn, 1996).

The World Vegetable Center identified two mungbean lines, V2709 and V2802, with complete resistance to bruchids; these lines are used to transfer the resistance to other cultivars (Talekar and Lin, 1981, 1992; Asian Vegetable Research and Development Center [AVRDC], 1991). Out of 500 mungbean accessions screened to *C. chinensis*, two entries V2802 B-G and V1128 B-BL were free from bruchid infestation. V2802 B-G is a selection from the resistant accession, V2802 (Asian Vegetable Research and Development Center [AVRDC], 1990b). Subsequently, four sets of straight or backcross progenies from V2709 or V2802 were screened. Out of 33 backcross progenies, only three progenies were free from bruchid infestation (Asian Vegetable Research and Development Center [AVRDC], 1992). Bruchid resistance in these resistant lines is controlled by a single dominant non-allelic gene (Asian Vegetable Research and Development Center [AVRDC], 1995). Line V2709 has been used as a bruchid-resistant source in Korea to develop bruchid-resistant variety Jangan (Hong et al., 2015), and in China to develop bruchid-resistant lines such as Zhonglv 3, Zhonglv 4, and Zhonglv 6 (Yao et al., 2015). However, Yao et al. (2015) in their 90-day subchronic oral toxicity study on rats found that daily doses of bruchid-resistant mungbean (derived from V2709) were well-tolerated. They did not observe any dose-related adverse effects in rats consuming diets formulated with bruchid-resistant mungbean compared with the bruchid susceptible cultivar diet and the AIN93G control diet. Two cultivated lines, V1128 and V2817, also showed resistance to *C. maculatus* (Somta et al., 2008).

Seventeen out of 525 *Vigna* accessions were found to be free from bruchid infestation at the World Vegetable Center (Asian Vegetable Research and Development Center [AVRDC], 1979). Of which, VM2011 and VM3529 were resistant, and VM2011 almost immune to bruchids. These two are the black gram (*Vigna mungo*) accessions. Also, a wild black gram (*V. mungo* var. *silvestris*) VM2164 has been used as a resistance source against bruchids (Asian Vegetable Research and Development Center [AVRDC], 1981; Sun et al., 2008; Souframanien et al., 2010). During 1981–82, mungbean breeder at the World Vegetable Center perfected the technique of crossing VM2011 and VM2164 with mungbean. In 1983, VM2164 was successfully crossed with several advanced breeding lines of mungbean and the resultant F_2_s and backcross progeny was screened. Twenty entries were free from bruchids and a mungbean accession, V4997, which was free of first generation adults, were selected for further screening (Asian Vegetable Research and Development Center [AVRDC], 1987). Subsequently, 101 mungbean breeding lines were screened. Five lines (VC1535-11-1-B-1-3-B, VC2764-B-7-2-B, VC2764-B-7-1-B, VC1209-3-B-1-2-B, and VC1482-C-12-2-B) showed least damage and at par with the resistant check, VM2164 (Asian Vegetable Research and Development Center [AVRDC], 1988). A bean fly resistant accession, V1160 (*V. glabrescens*) also showed higher resistance to bruchids (Asian Vegetable Research and Development Center [AVRDC], 1990a). However, screening of backcross progenies of the cross involving V1160 and a high yielding, bruchid susceptible mungbean accession (VC1973A) showed that all the progenies were susceptible to bruchids (Asian Vegetable Research and Development Center [AVRDC], 1990c).

Although many sources of resistance to bruchids have been identified in *Vigna* subgenus *ceratotropics*, most of them are wild species belonging to the Angulares group and are cross-incompatible with mungbean, which leads to the incorporation of undesirable traits (Tomooka et al., 1992, 2000). Presently, mungbean breeders and entomologists have only few mungbean accessions, which include TC1966, ACC41, V2709, V2802, V1128, V2817, ACC23 and Indian sublobata (Sub2) as sources of resistance against bruchids (Somta et al., 2007, 2008; Mei et al., 2009; Sarkar et al., 2011). Although Sarkar et al. (2011) reported bruchid resistance in IC333175, IC325770, IC329039, Dantan Sonamung, RS4, RMG11 and Khargone1, their use as the source to develop bruchid resistance mungbean needs to be identified. Genetic resistance to bruchids has also been identified in cowpea, wild black gram (*V. mungo* var. *silvestris*), rice bean (*V. umbellata*) and wild relatives of azuki bean (*V. napalensis*) (Dongre et al., 1996; Tomooka et al., 2000).

## Host Plant Resistance

Association of bruchids and leguminous plants is a co-evolutionary process and both have evolved precisely to avoid the defensive systems of the each other. In this arms race, legumes have produced many toxic compounds to kill or deter bruchids. The bruchids, in turn, have developed adaptive strategies to combat the effect of these toxic compounds. The interactions between bruchids and legumes are highly specific, as one insect species feeds on a very few seed species (Somta et al., 2007). Host plant resistance against insect pests is manifested through antibiosis, antixenosis (non-preference) and/or tolerance (Talekar and Lin, 1992; Edwards and Singh, 2006). The resistant traits can be morphological, physiological and/or biochemical, and affect growth and development of insect pests (Talekar and Lin, 1992; Lattanzio et al., 2005; Edwards and Singh, 2006). The morphological traits in legumes include color and shape of the pod and seed, while the physiological and/or biochemical traits include secondary metabolites and anti-nutritional compounds affecting the metabolic activity of bruchids (Sarikarin et al., 1999; Appleby and Credland, 2003; Lattanzio et al., 2005; Somta et al., 2007). In next few sections, we will discuss various forms of resistance evolved in mungbean against bruchids and counter-adaptations in bruchids (if any) against them.

## Physical Basis of Resistance

The first encounter between insect pests and host plants is oviposition by insect pests; the pests’ preference or non-preference determines the resistance and/or susceptibility of the host plants. Successful oviposition is necessary for successful population build-up and high infestation. Any adverse effect on insect oviposition will have detrimental effects on the subsequent pest population build-up. Thus the suitability of the host plant surface for insect oviposition will show how good it is for the progeny’s survival and development.

A number of antixenotic traits are implicated by plants to avoid insect oviposition in both field plants and storage seeds (Watt et al., 1977; Petzold-Maxwell et al., 2011; War et al., 2013). These traits determine the host plant/seed resistance or susceptibility to oviposition and include surface chemicals, plant volatiles, spines, hairs, etc. (Watt et al., 1977; Petzold-Maxwell et al., 2011; War et al., 2013). The host plant/seeds avoid insect oviposition either directly or indirectly by killing the insect eggs to avoid hatching of the larvae, thus, preventing future damage (Doss et al., 2000; Petzold-Maxwell et al., 2011). Traits contributing to resistance/susceptibility of mungbean to bruchids include seed color, texture, hardness, size and chemical constituents (Asian Vegetable Research and Development Center [AVRDC], 1979, 1981; Sarikarin et al., 1999; Appleby and Credland, 2003; Lattanzio et al., 2005; Somta et al., 2007). Seed texture of legumes affects the oviposition capacity of *C. maculatus* and *C. chinensis* (Sarikarin et al., 1999). Female bruchids prefer to lay eggs on smooth surface seeds rather than rough surface seeds covered with an inner pod membrane that renders the seed dull (Watt et al., 1977). Fujii et al. (1989) observed that seed of the bruchid-resistant mungbean TC1966 is covered with a network of parallel and transverse ridges, unlike the smooth surface of commercial mungbean. This characteristic makes the female bruchid rather hesitant to lay eggs on the seed. Dense hairs on the pods of VM2011 and VM3529 make it difficult for the adult bruchids to move over the pods and decrease the number of eggs laid and adult emergence from pods (Asian Vegetable Research and Development Center [AVRDC], 1979, 1981). The highly resistant VM2164 seems to have hard seed coat (Asian Vegetable Research and Development Center [AVRDC], 1981). However, some reports rule out any role of seed coat in bruchid resistance in mungbean (Somta et al., 2006, 2008). Seed size may also affect oviposition preference of bruchids and a strong correlation has been observed between bruchid resistance and small- or medium-sized seeds (Lambrides and Imrie, 2000; Somta et al., 2007; Mei et al., 2009). Small-sized seeds show more resistance to bruchids than the medium or large seeds (Lambrides and Imrie, 2000). They suggested that resistance to *C. chinensis*, *C. maculatus* and *C. phaseoli* in wild mungbean accessions ACC23 and ACC41 (*V. radiata* subsp. *sublobata*) could be attributed to the thick texture layer present on the seeds of these accessions, which might have acted as an oviposition deterrent to bruchids. The association between bruchid resistance and seed size has been further supported genetically by co-location of the quantitative trait loci (QTLs) for bruchid resistance and seed mass (Mei et al., 2009), but this does not rule out the effect of environmental factors on the interactions between seed size and bruchid resistance. Contrasting results have been shown by Tomooka et al. (2000), where no relationship was observed between seed size and bruchid resistance in 20 *Vigna* species. Thus, the morphological traits such as seed coat, seed smoothness/roughness, pod hairiness, and seed shine/dullness could form important morphological markers in plant breeding for developing bruchid-resistant mungbean.

## Biochemical Basis of Resistance

Plant toxic secondary metabolites are important defensive traits involved in plant defense against insect pests (Birch et al., 1986; Asian Vegetable Research and Development Center [AVRDC], 1992; Chen et al., 2002; Wisessing et al., 2008; War et al., 2012, 2013). They act either directly on insect pests through antibiosis or develop the non-preference for insects feeding on the seeds (War et al., 2012, 2013). An array of compounds found in seeds acts either additively or synergistically against insect pests including bruchids. These include naringenins, vicilins, cysteine-rich protein (VrD1 or VrCRP), vignatic acids (A and B) and *para*-amino- phenylalanine (Birch et al., 1986; Sugawara et al., 1996; Chen et al., 2002; Somta et al., 2007). V2709, V2802, and VM2164 possess high antibiosis mechanisms of resistance due to the toxic secondary metabolites (Asian Vegetable Research and Development Center [AVRDC], 1992). Resistance-related proteins such as chitinase, β-1,3-glucanase, and peroxidase are also involved in the bruchid resistance in mungbean (Khan et al., 2003). However, the bad taste or toxicity of these chemicals to non-target organisms has posed a great challenge to scientists to minimize their effects but to stabilise the resistance. VrD1 protein (*V. radiata* defensin 1, previously named VrCRP) is a cysteine-rich protein isolated from seeds of VC6089 that imparts resistance to mungbean against *C. maculatus* (Chen et al., 2002). It exhibits insecticidal as well as growth-inhibitory effects against *C. maculatus* even at a concentration of 0.1% (Lin et al., 2005). Further, its activity at 0.2% (wt:wt) has been found to be higher than amylase inhibitor (0.5%), vignatic acid A (1.0%) and some specific lectins (2.0%) (Murdock et al., 1990; Sugawara et al., 1996). It has been suggested that VrD1-based transgenic crops or VrD1 based bio-insecticides could form an important component of bruchid management programs (Lin et al., 2005).

Mungbean seeds contain lignins, quinines, alkaloids, saponins, non-protein amino acids and polysaccharides, and anti-nutritional seed proteins such as lectins, phytohemagglutinins (PHA), and proteinase inhibitors involved in resistance against bruchids (Landerito et al., 1993; Lattanzio et al., 2005; Wisessing et al., 2008). Trypsin inhibitors have been recorded in higher levels in bruchid-resistant varieties in mungbean than the susceptible ones (Landerito et al., 1993). The α-amylase inhibitors interfere with bruchid digestive enzymes and can act as a biocontrol agent against them (Wisessing et al., 2008). Plants utilise them against a variety of insect pests belonging to Coleoptera, Homoptera, Diptera, and Lepidoptera (Macedo et al., 2007; Vandenborre et al., 2011; War et al., 2012).

Plant lectins are carbohydrate-binding (glyco) proteins that reversibly bind to well-defined simple sugars and/or complex carbohydrates (Vandenborre et al., 2011; War et al., 2012). The insecticidal property of lectins has been attributed to their survival in the digestive system of herbivores (Vandenborre et al., 2011; War et al., 2012). They bind to the glycosyl groups lining the digestive tract, are pH stable, and interfere with digestion and absorption in the insect gut (Vandenborre et al., 2011). In legumes, lectins are accumulated in seeds and provide a potential defense against seed infesting insect pests, especially bruchids (Oliveira et al., 1999). Canavalin (vicilin, 7S globulin) in the seed coat has detrimental effects on the development of bruchids (Oliveira et al., 1999). Two major D-galactose-specific lectins (MBL-I and MBL-II) have been characterised from mungbean seeds (Suseelan et al., 1997), but their role in bruchid resistance has not been studied. Lectins from various plants have been reported to alter the growth and development of bruchids (Leite et al., 2005; Macedo et al., 2007; War et al., 2012). For example, lectins from the leaves of *Bauhinia monandra* Kurz (BmoLL) showed insecticidal activity against *C. maculatus*, when provided with artificial seeds (Macedo et al., 2007). Lectins such as Canatoxin from *Canavalia ensiformis* (L.), Zeatoxin from *Zea mays* seeds, seed lectin from *Talisia esculenta* Radlk., galactose-specific lectin from African yam beans, *Sphenostylis stenocarpa* (Hochst. ex A. Rich.) and a lectin from the marine red alga, *Gracilaria ornata* Areschoug, have been found highly toxic to *C. maculatus* (Oliveira et al., 1999; Leite et al., 2005; Macedo et al., 2007). They bind to the midgut proteins and reduce the α-amylase activity of *C. maculatus* larvae (Macedo et al., 2007). Accumulation of cyanogenic glycosides and phytic acid in mungbean seeds during seed maturation plays an important role in defense against bruchids (Lattanzio et al., 2005). Vignatic acid A confers bruchid resistance in mungbean wild variety TC1966 (Sugawara et al., 1996). These lines also contain a peptide compound (GIF-5) and a cysteine-rich protein (VrCRP) in seed coats that impart resistance against bruchids (Chen et al., 2002). About 0.2% VrCRP in mungbean seeds completely inhibit larval development in bruchids (Chen et al., 2002). Further, albumin content in mungbean lines affects egg laying in bruchids, increases larval developmental periods, and reduces adult longevity (Landerito et al., 1993). Bruchid resistance in VC6089A, TC1966, and the recombinant inbred line 59 (RIL59) has been attributed to the resistant-specific protein, gag/pol polyprotein, and aspartic proteinase (Lin et al., 2016).

Although the biochemical defenses utilized by legumes against bruchids are effective, bruchids have developed counter-adaptations to most of these toxic chemicals (Murdock et al., 1988; Zhu-Salzman et al., 2003; Ahn et al., 2004; Chi et al., 2009). The counter-adaptations in bruchid toward mungbean defensive traits have not been studied in detail, however, the adaptations of these pests to the defensive traits of the closely related legumes such as cowpea shows the possibility that bruchids could adapt to the mungbean defense system as well. These adaptations would have a major bearing on bruchid resistance in mungbean. Bruchids have evolved metabolic pathways to bypass the enzyme block. Protein anti-metabolites such as proteinase inhibitors, lectins and α-amylase inhibitors are governed by a single gene and there is every possibility that bruchids could adapt to them by producing high levels of mid-gut aspartic and cysteine proteinase (Silva et al., 1999; Zhu-Salzman et al., 2003). The major digestive cathepsin L-like cysteine proteases in bruchids are CmCPA and CmCPB (Koo et al., 2008). When fed on a diet containing soybean cysteine protease inhibitor N (scN), *C. maculatus* expressed high levels of CmCPB to neutralize the effect of protease inhibitors (Ahn et al., 2004). Furthermore, the scN is hydrolyzed by aspartic proteases, which are further degraded by cysteine and serine proteases (Zhu-Salzman et al., 2003; Ahn et al., 2004). Bruchids fed on scN-based diet showed the regulation of a large number of genes that are involved in counter defense and stress responses (Moon et al., 2004; Chi et al., 2009). In *C. maculatus*, the expression of scN-insensitive CmCatB occurs through the regulation of positive HNF-4 and negative CmSvp factors (Zhu-Salzman et al., 2003). Bruchids have developed adaptation to toxic compounds by the over-production of various enzymes including glutathione *S*-transferases, cytochrome P450 monooxygenases (P450s) and esterases (Zhu-Salzman et al., 2003; Ahn et al., 2004, 2007; Chi et al., 2009). Some molecular understanding of bruchid adaptations to plant secondary metabolites has been carried out but the upstream regulatory mechanism(s) that induce the transcriptional and post-transcriptional regulation in bruchids when fed on legumes including mungbean is limited. This deserves in-depth studies to unravel the mechanisms that could be regulated to overcome bruchid adaptation to legumes such as mungbean secondary metabolites.

## Genetic Basis of Resistance

The resistant genes against various bruchid species in mungbean have been reported in wild species such as TC1966 (*V. radiata* var. *sublobata*) (Fujii and Miyazaki, 1987; Fujii et al., 1989; Lambrides and Imrie, 2000; Kashiwaba et al., 2003). Plant breeders have been using this variety to transfer the resistant genes to develop bruchid-resistant mungbean cultivars (Talekar, 1988; Tomooka et al., 1992; Somta et al., 2007). Bruchid resistance has also been reported in several closely related wild *Vigna* species including wild black gram (*V. mungo* var. *silvestris*) (Tomooka et al., 2000; Pandiyan et al., 2010; Sharma et al., 2013). Bruchid resistance in mungbean has been mapped using restriction fragment length polymorphism (RFLP), random amplified polymorphic DNA (RAPD), simple sequence repeat (SSR), and single nucleotide polymorphism (SNP) markers (Young et al., 1992; Menancio-Hautea et al., 1993; Villareal et al., 1998; Chen et al., 2007; Chotechung et al., 2011). In TC1966, 14 linkage groups containing 153 RFLP markers were mapped having 9.3 centiMorgans (cM) as an average distance between the markers (Young et al., 1992). They identified an F_2_ population individual from a cross between TC 1966 and a susceptible cultivar retaining the bruchid resistance gene within a tightly linked double crossover and were further utilized for developing the bruchid-resistant mungbean lines without any linkage drag. The genetic localization of bruchid resistance traits such as cyclopeptide alkaloids and vignatic acid was studied by Kaga and Ishimoto (1998). Menancio-Hautea et al. (1993) constructed an RFLP linkage map of mungbean, where the bruchid resistance gene is located at the 13 cM interval and is flanked by RFLP markers. A genetic linkage map of mungbean was constructed from a cross-derived population from the mungbean cultivar ‘Berken’ and a wild mungbean genotype ACC41 using RFLP (Humphry et al., 2002). The first mungbean bacterial artificial chromosome (BAC) libraries with two polymerase chain reaction-based markers *STSbr1* and *STSbr2*, analyzed against a RIL population between ACC41 (resistant line) and Berken (susceptible line), are closely linked with a major locus conditioning bruchid resistance (Miyagi et al., 2004). Similar markers were used by Sarkar et al. (2011) to validate a *V. sublobata* accession (sub2) and 12 other cultivars for bruchid resistance, where *STSbr1* amplified a 225 bp fragment in all the resistant plants. Recently, Wang et al. (2016) constructed the linkage groups of mungbean and mapped the Br1 locus using the RIL population from the cross between Berken and ACC41. They showed Br1 on LG9 between BM202 (0.7 cM) and Vr2-627 (1.7 cM). Chen et al. (2007) constructed a linkage map for *Br* and the vignatic acid gene (*Va*) using RFLP and identified eight RAPD markers linked to the *Br* gene. Markers for bruchid resistance have also been identified by RAPDs and utilized in conjunction with an RIL and near-isogenic line (NIL) mapping population (Villareal et al., 1998) using TC 1966. The NILs used included B4P3-3-23, B4P 5-3-10, B4Gr3-1 and DHK 2-18, and carried the genes resistant to bruchids in four genetic backgrounds such as Pagasa 3, Pagasa 5, Taiwan Green and VC 1973A, respectively. Chen et al. (2007) developed 200 RILs involving bruchid resistance in TC1966 and mungbean yellow mosaic virus resistant variety NM92 and identified 10 RAPD markers associated with bruchid resistance through bulked segregant analysis, of which four (OPW02, UBC223, OPU11, and OPV02) were closely linked. Seven codominant cleaved amplified polymorphisms developed from the identified RAPD markers showed tighter linkage with the *Br* gene than the original RAPD. The mungbean SSR marker DMB-SSR 158 is perfectly associated with bruchid resistance in V2802 (Chotechung et al., 2011) and TC1966 (Chen et al., 2013) with the distance of <0.1 cM between DMB-SSr 158 and the major QTL in TC1966 (Chen et al., 2013). On chromosome 5, this marker is associated with polygalacturonase inhibitor genes (*Vradi05g03940-VrPGIP1* and *Vradi05g03950-VrPGIP2*) that account for resistance to bruchids (Chotechung et al., 2016). They concluded that the gene *VrPGIP1* could be the candidate gene for bruchid resistance in mungbean. Lin et al. (2016) observed that bruchid resistance in a NIL VC6089A occurs due to the BURP (BNM2, USP, RD22, and PG1β) protein family. They further observed the higher expression of g39185 (resistant-specific protein), g34458 (gag/pol polyprotein), and g5551 (aspartic proteinase) in bruchid-resistant lines (VC6089A, TC1966, and RIL59) than the susceptible ones (VC1973A and NM92), which could be implicated for screening bruchid resistance/susceptibility in mungbean.

Although bruchid-resistant mungbean retains the transcript diversity and specificity by differentially expressed genes (DEGs) and sequence-changed protein genes (SCPs) (Liu et al., 2016), it has not been confirmed if all the identified DEGs and SCPs confer resistance against bruchids. Studies based on QTL revealed that a major Br locus and a few minor loci with one or two genes might account for bruchid resistance in mungbean (Kitamura et al., 1988; Young et al., 1992; Chen et al., 2013). Hong et al. (2015) identified two QTLs located between MB87 and SOPU11 for bruchid resistance in V2709. In ACC41, a QTL accounting for about 98.5% of bruchid resistance was identified by Mei et al. (2009). The mungbean populations derived from TC1966 and V2802 carry a strong QTL locus on chromosome 5 for bruchid resistance, suggesting that they carry the same QTL for bruchid resistance with a strong linkage in co-segregating alleles (Schafleitner et al., 2016). Further, the accuracy of the identified molecular markers in predicting resistance and susceptible genotypes is about 100%. The resistance locus to bruchids in ACC41 and TC1966 could be same as the F_2_ population from the crosses of the two did not show any susceptible segregate (Mei et al., 2009). The important points to use QTLs in breeding include QTL confirmation, QTL validation and/or fine (or high resolution) mapping. Tremendous efforts have been made in mapping bruchid-resistant genes by RFLP and RAPD marker constructed linkage maps (Kaga and Ishimoto, 1998), and for studying genetic diversity in mungbean by RAPD along with inter simple sequence repeat profiles (Chattopadhyay et al., 2005). The RAPD was suggested as a fast and simple molecular marker technique in identifying bruchid resistance in mungbean, however, the markers are far away from the resistant gene. Based on the genome size and the length of linkage groups in mungbean, the tightly linked markers can be effectively used in marker assisted selection, fine mapping, and gene cloning. However, further in-depth investigations are needed in this area for developing the stable bruchid-resistant mungbean.

## Breeding Constraints for Developing Bruchid-Resistant Mungbean

Breeding mungbean for resistance to bruchids is a complex and laborious process and takes several generations of backcrossing to build up a fixed line with complete resistance. For obtaining a perfect stable mungbean cultivar, bruchid-resistant must be combined with consistent high yield, seed structure and size, seed quality and nutritional value, plant type and the desired maturity. It has been shown that in mungbean, bruchid resistance is controlled by a single gene (Humphry et al., 2002; Miyagi et al., 2004; Sun et al., 2009). To achieve bruchid resistance in mungbean without compromising on agronomic traits is a technical challenge being faced by the plant breeders (Keneni et al., 2011), since undesirable genetic linkages impede the proper exploitation of genetic diversity from wild germplasm into the commercial cultivars (Edwards and Singh, 2006; Acosta-Gallegos et al., 2008). Further, co-inheritance of the undesired and desired traits may reduce seed quality, making them unfit for consumption, and may also cause a reduction in yield (Keneni et al., 2011). Nevertheless, genetic drag has a major bearing on the expression of bruchid resistance in mungbean when the pest-resistant traits are controlled by many genes and are of low dominance, resulting in passing of many undesirable traits such as differential leaf size, seed texture, and color along with the desired traits during breeding (Edwards and Singh, 2006). However, crossing over between homologous chromosomes during meiosis can break the linkage between the genes for desired and undesired traits. Thus growing a large number of F_2_ populations to increase recovery of new recombinants due to crossing-over is required. Further, backcrossing is an important approach that could reduce the undesired effects of linkage drag (Keneni et al., 2011).

Biotypic variation, i.e., genetic variability of the pest population, is one more challenge for the breeders. Development of biotypes has led to the breakdown of resistance in mungbean against bruchids (Fox et al., 2010). A cultivar resistant to one biotype may be susceptible to another, and the development of a cultivar with resistance against multiple biotypes is a complicated process. Although TC1966 has been reported as resistant to all the bruchid biotypes, the cultivars need to be screened for such interactions. There could be the possibility of the cultivars showing different levels of resistance/susceptibility to bruchid biotypes. Also, a crop cultivar can at the same time be exposed to more than one biotypes. To develop cultivars with durable resistance to all the possible biotype combinations would be highly challenging for the plant breeders. Gene pyramiding could play an important role in tackling resistance against bruchid biotypes by the incorporation of multiple resistant genes in a cultivar. Also, lack of interspecific cross-compatibility is an important issue in breeding mungbean for resistance against bruchids. Transferring pest-resistant genes from wild species of related legumes such as black gram to cultivars for bruchid resistance in mungbean could show interspecific cross-incompatibility (Keneni et al., 2011). However, some reports showed successful hybridisation between black gram and mungbean (Pandiyan et al., 2010; Sharma et al., 2013).

The process of identification of the segregating populations of early and late generations by artificial bioassays and biochemical methodologies for resistance against bruchids is expensive, time- consuming, and demands significant resources and technical specificities. Indirect selection for traits such as seed texture, seed size etc., that confer resistance against bruchids in mungbean cannot be consistent (Somta et al., 2007; Srinivasan and Durairaj, 2007), and environmental factors would affect the resistance and/or susceptibility of the cultivars against bruchids (Appleby and Credland, 2004). Bruchid resistance studies of mungbean seeds showed that diploid maternal tissue gives rise to seed coat and progeny tissue forms in the diploid embryo and the triploid endosperm (Somta et al., 2007).

All the breeding entities involving morphological, biochemical and molecular markers form valuable resources that could be used together to mitigate the breeding constraints in developing bruchid resistance mungbean.

## Future Outlook

Bruchid resistance in mungbean has attained considerable momentum and has attracted the attention of plant breeders, entomologists, and biotechnologists worldwide, but many resistance-related issues have yet to be unravelled. Although some sources of resistance have been identified and are being used to modify the gene pool of commercial cultivars to develop bruchid-resistant mungbean, undesired characters may be pronounced in the insect-resistant cultivar. Studies on bruchid- resistance in relation to the development of molecular markers have gained high momentum (Kitamura et al., 1988; Chen et al., 2007). The molecular markers for bruchid-resistance have increased the selection efficiency and reduced the number of selection tests as well as the cost required for screening (Kitamura et al., 1988; Chen et al., 2007; Schafleitner et al., 2016). This has minimized the dependence on phenotypic data (Kitamura et al., 1988; Chen et al., 2007). Further, bruchid resistance from black gram can be transferred into mungbean efficiently using interspecific or intraspecific crosses supported by bruchid resistance gene markers (Pandiyan et al., 2010; Sharma et al., 2013; Kim et al., 2015). RFLP markers have been widely used for mapping the bruchid-resistant gene in mungbean, owing to its complicated protocol, however, this marker system has not been practical for marker assisted selection. SSR markers could be the better option, but their numbers are limited in mungbean and have not been widely used. Nevertheless, due to the evolutionary pressure, bruchids may adapt to the newly formed resistant lines with single-gene resistance; thus, there is a need to identify and combine multiple resistant genes into the same cultivar. The biochemical traits imparting seed resistance against bruchids in new sources of resistance need to be identified and studied thoroughly to see if there is any toxic effect on animals and/or any other undesirable effects on natural enemies of insect pests. In addition, herbivore-specific signal molecules, their identification, mode of action, and further signal transduction needs to be studied. Because a single attribute can affect herbivores and natural enemies positively or negatively, it is important to understand the multi-trophic interactions and the consequences of supposed defensive traits of a plant for use in pest management. An approach that could combine defensive pathways in mungbean and the counter-adaptive pathways in bruchids can be successfully used to develop bruchid-resistant mungbean.

## Author Contributions

AW, SM, VB, RN, and RS wrote the manuscript. AW, RN, and RS revised it.

## Conflict of Interest Statement

The authors declare that the research was conducted in the absence of any commercial or financial relationships that could be construed as a potential conflict of interest.
